# Is it becoming harder to secure reviewers for peer review? A test with data from five ecology journals

**DOI:** 10.1186/s41073-016-0022-7

**Published:** 2016-11-04

**Authors:** Arianne Y. K. Albert, Jennifer L. Gow, Alison Cobra, Timothy H. Vines

**Affiliations:** 1 Women’s Health Research Institute, 4500 Oak Street, Vancouver, BC Canada V6H 3N1; 2 Molecular Ecology Editorial Office, 6270 University Blvd, Vancouver, BC Canada V6T 1Z4; 3 Current address: Axios Review Editorial Office, 4521 John Street, Vancouver, BC Canada V5V 3X3

**Keywords:** Peer review, Reviewers, Academic journals

## Abstract

**Background:**

There is concern in the academic publishing community that it is becoming more difficult to secure reviews for peer-reviewed manuscripts, but much of this concern stems from anecdotal and rhetorical evidence.

**Methods:**

We examined the proportion of review requests that led to a completed review over a 6-year period (2009–2015) in a mid-tier biology journal (*Molecular Ecology*). We also re-analyzed previously published data from four other mid-tier ecology journals (*Functional Ecology*, *Journal of Ecology*, *Journal of Animal Ecology*, and *Journal of Applied Ecology*), looking at the same proportion over the period 2003 to 2010.

**Results:**

The data from *Molecular Ecology* showed no significant decrease through time in the proportion of requests that led to a review (proportion in 2009 = 0.47 (95 % CI = 0.43 to 0.52), proportion in 2015 = 0.44 (95 % CI = 0.40 to 0.48)). This proportion did decrease for three of the other ecology journals (changes in proportions from 2003 to 2010 = −0.10, −0.18, and −0.09), while the proportion for the fourth (*Functional Ecology*) stayed roughly constant (change in proportion = −0.04).

**Conclusions:**

Overall, our data suggest that reviewer agreement rates have probably declined slightly but not to the extent suggested by the anecdotal and rhetorical evidence.

## Background

There is a widespread perception among Editors of academic journals, as well as the broader research and scholarly publishing communities, that it is becoming harder and harder to find willing reviewers ([1, 7, 11, 12], Baveye and Trevors 2011 [2], [8, 9, 13]). The most common reason cited is “reviewer fatigue,” whereby the steady increase in submissions to journals in the past two decades has meant that ever more review requests must be sent to the same limited pool of reviewers [3, 18]. Other factors contributing to fatigue include the falling success rates of funding applications [14] and the increase in other administrative tasks expected from academics [10]. However, despite the certainty that normally accompanies statements about the increasing difficulty of finding reviewers, there have been very few attempts to quantify the severity of this problem with journal data (for an exception, see [5]).

The peer review system is consistently ranked by researchers as an important function of journals [4], [15]. A significant decline in the willingness of researchers to contribute reviews would be a serious problem for scholarly publishing. Even in the short term, a reduced reviewer agreement rate would add significantly to the time it takes to find sufficient reviewers, lengthening the delay between manuscript submission and the editorial decision.

With the exception of Fox et al. [5], who estimated a reduction of reviewer agreement rates from about 70 % in 2004 to about 45 % in 2014 for *Functional Ecology*, there have been no published attempts to quantify the reviewer acceptance and completion rates at other journals. Therefore, to add to the Fox et al. [5] results, we conducted a similar analysis on the rate of review completion in *Molecular Ecology* from 2009 to 2015. We also re-analyzed publicly available data from four other ecology journals (including *Functional Ecology*; [16], [5, 6]) to investigate if there is a larger trend among journals. As a second hypothesis, we also tested whether it was harder to find reviewers for lower quality papers at *Molecular Ecology*.

## Methods

### Molecular Ecology

We analyzed new submissions of original articles (the most common article type) to *Molecular Ecology* in each of the following years: 2009, 2011, 2013, and 2015. Only original submissions that went out for a first round of peer review were included in the dataset; papers that were resubmitted to the journal for a second round of review were excluded, as were papers intended for publication in a special issue. We selected all papers meeting the above criteria starting from October 1st in each year until 100 papers had been collected per year. The 100th paper was submitted in mid to late November in all years. These data were collected manually from the online system used to manage peer review at *Molecular Ecology* (ScholarOne Manuscripts, provided by Thomson Reuters). ScholarOne allows for several different responses to an emailed review request. These were

- Declined: this included reviewers who actively declined as well as those who did not respond to the invitation or subsequent reminders. This response was also used when we could not find a valid email for the reviewer in 2009, 2011, and 2013.
- Unassigned: this covered reviewers who accepted an invitation but were later unassigned i.e., they did not complete a review. This response category was included in the total review request count.
- Uninvited: invitations that were revoked before a reviewer could respond, typically because the editorial office spotted an error or had sufficient reviews to make a decision. Since these do not allow for an accept/decline choice by the reviewer, these were excluded from the total review request count.
- Completed: reviewers who returned a review after accepting an invitation.

The additional category “Invalid Email” was introduced in 2014 to denote review requests that generated an email error. Since this response type was included with “Declined” in previous years, we combined the “Invalid Email” response with “Declined” for the 2015 manuscripts.

To test whether it was harder to find reviewers for lower quality papers, we approximated the quality of the paper with the editorial decision. This approach would be less accurate if high-quality papers were routinely reviewed then rejected for being out of scope. Since decisions of this kind were extremely rare at *Molecular Ecology* (out of scope papers were typically returned to the authors without being reviewed), we believe that the editorial decision is a fair reflection of the paper’s overall quality. The decision type categories were as follows:

- Reject: the paper was rejected outright.
- Reject, encourage resubmission (REnc): the paper was rejected, but the authors were encouraged to resubmit a new version of the paper for a second round of peer review.
- Reconsider after revision (RAR): the paper is provisionally accepted, conditional on the new version adequately addressing requested revisions. The revised version would be re-assessed by the Associate Editor.
- Minor revisions (MRev): the paper is provisionally accepted with a request to make some additional changes. The revised version would be assessed by the Managing Editor.

We conducted a logistic regression of the proportion of completed reviews out of all review invitations for *Molecular Ecology*, with year and editorial decision included as independent variables in the model. Significance of the variables was assessed using likelihood ratio tests that compared the deviance of the model containing the variable vs. the deviance of the model with that variable removed.

### Other ecology journals

Petchey et al. [16] conducted an analysis of the relationship between the number of manuscripts submitted by a researcher and the number of papers they reviewed. They accessed data on articles submitted and review requests sent between 2003 and 2010 for four ecology journals owned by the British Ecological Society (BES): *Journal of Animal Ecology*, *Journal of Ecology*, *Journal of Applied Ecology*, and *Functional Ecology*. These datasets were collected using ScholarOne Manuscripts reporting system and are available on Dryad (http://datadryad.org/resource/doi:10.5061/dryad.36r69, Petchey et al. 2014b). The “authors” subset of these datasets gives the number of review requests sent and the number of reviews returned for each reviewed submission at each journal, along with the editorial decision. The data included both original submissions and resubmitted manuscripts (Lindsay Haddon, pers. comm.), although these could not be distinguished in the dataset.

Before re-analyzing this dataset, we removed papers where the number of review requests was zero, and the single instance where the number of reviews obtained was higher than the number of requests sent. We did not conduct a logistic regression on the proportion of completed reviews for these journals. This was because the number of papers in each journal in each year was very large (~1000), so that even small differences among years would likely have been statistically significant without necessarily being significant in practice. Therefore, we calculated average proportions per year with 95 % confidence intervals and used those to make comparisons among years. The decision categories at all four journals changed through time and were unclear as to their relative hierarchy. Therefore, we did not use final editorial decision in this comparison.

The results in Fox et al. [5] are split by gender of the invited reviewer. Since the data for Fox et al. [5] is publicly available (http://dx.doi.org/10.5061/dryad.5090r, [6]), we were able to calculate an overall review request acceptance rate that is roughly comparable to the review completion rate for the other journals examined here.

## Results

### Molecular Ecology

**Fig. 1:**
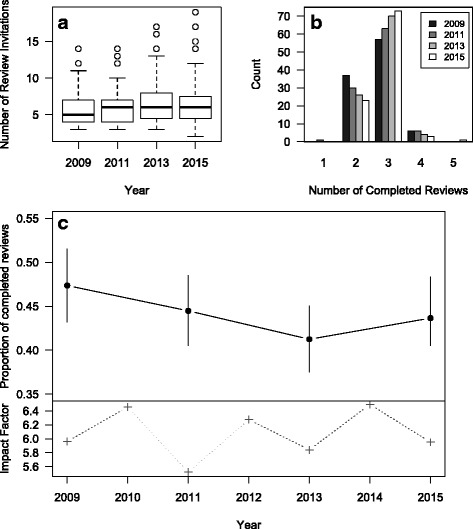
*Molecular Ecology* data, plotted by year. **a** Distribution of the number of review invitations sent between 2009 and 2015 by year. The *black lines* are the medians, the *boxes* indicate the interquartile range, the *whiskers* extend to 1.5 times the interquartile range (IQR), and the *points* are outliers beyond 1.5*IQR. **b** Number of manuscripts with different numbers of completed reviews by year. **c** Mean proportion of completed reviews by year with the bottom panel showing the impact factor in *grey crosses*. The *error bars* indicate the 95 % confidence intervals

**Fig. 2:**
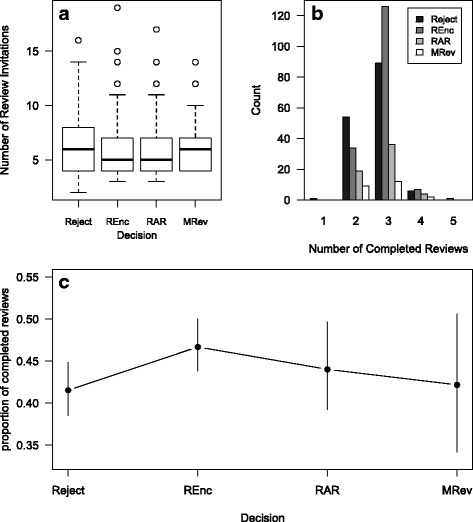
*Molecular Ecology* data, plotted by editorial decision type. **a** Distribution of the number of review invitations sent for manuscripts with different editorial decisions. The *black lines* are the medians, the *boxes* indicate the interquartile range, the *whiskers* extend to 1.5 times the interquartile range (IQR), and the *points* are outliers beyond 1.5*IQR. **b** Number of manuscripts with different numbers of completed reviews by decision type. **c** Mean proportion of completed reviews by decision. The *error bars* indicate the 95 % confidence intervals

The logistic regression found no significant relationship between either year (likelihood ratio test statistic = 4.5, *p* = 0.21; Fig. 1) or decision (likelihood ratio test statistic = 6.6, *p* = 0.09; Fig. 2) and the proportion of completed reviews. This suggests that the success rate of review requests is not different among years or among papers with differing editorial decisions.

The average number of review requests per paper sent to reviewers increased slightly between 2009 and 2015 (Table 1 and Fig. 1). However, the number of completed reviews also increased, such that the proportion completed remained more or less the same (proportion in 2009 = 0.47 (95 % CI = 0.43 to 0.52), proportion in 2015 = 0.44 (95 % CI = 0.40 to 0.48); Fig. 1).

**Table 1:** Summary of reviewer invitations and responses

	Mean number of reviews per manuscript:					Mean proportion of reviews completed (95 % CI)
Year	Invited	Declined	Unassigned	Uninvited	Completed	
2009	5.68	2.76	0.23	0.14	2.69	0.47 (0.43 to 0.52)
2011	6.16	3.18	0.24	0.08	2.74	0.44 (0.41 to 0.49)
2013	6.74	3.77	0.19	0.05	2.78	0.41 (0.38 to 0.45)
2015	6.46	3.43	0.10	0.02	2.82	0.44 (0.40 to 0.48)

The table includes only original submissions that went out for peer review. Categories are invited (all reviewers who were invited regardless of whether they accepted and completed a review, declined, had an invalid email, or were unassigned); declined (reviewers who actively declined as well as those who did not respond, and those with invalid emails); unassigned (reviewers who accepted an invitation but were later unassigned, i.e., did not complete a review); uninvited (reviewers whose invitations were revoked before they responded); and completed (reviewers who returned a review after accepting an invitation). The last column gives the mean proportion of review invitations that lead to a review being completed

When the *Molecular Ecology* data are broken down by the editorial decision, there is no clear trend for one type to have received more invitations. Similarly, the manuscripts tended to have a similar proportion of reviews completed regardless of the type of decision (Fig. 2).

### Other ecology journals

**Fig. 3:**
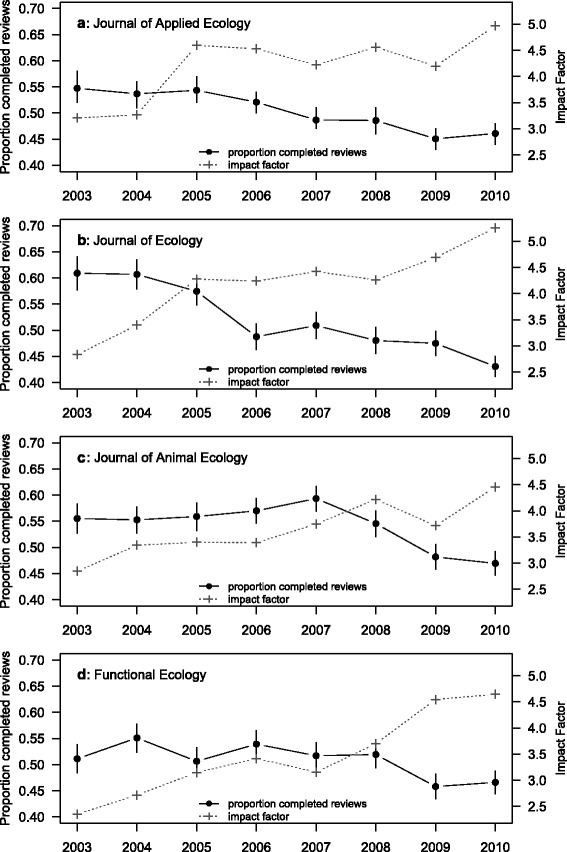
Proportion of review requests that lead to a completed review for four ecology journals (**a**, **b**, **c**, **d**), 2003 to 2010 shown in *solid circles*. Data from [17]. *Error bars* indicate 95 % confidence intervals. *Right-hand* axis shows the impact factor for each journal (*grey crosses*)

Trends in the proportion of review requests that led to a completed review varied among the other four ecology journals (Fig. 3). There was a fairly linear reduction in the proportion of completed reviews in the *Journal of Applied Ecology*, with the proportion changing from 0.56 (95 % confidence intervals: 0.52 to 0.58) in 2003 to 0.46 (0.44 to 0.48) in 2010 (Fig. 3). Similarly, the *Journal of Ecology* and *Journal of Animal Ecology* both had overall reductions in the proportion of completed reviews from 2003 to 2010 (from 0.61 to 0.43 and 0.56 to 0.47, respectively). However, these reductions appeared to be less linear, and the journals had several years with more or less the same proportion followed by a sharp reduction (Fig. 3). *Functional Ecology*, in contrast, showed no convincing evidence for a decrease in the proportion of completed reviews, from 0.51 (0.48 to 0.54) in 2003 to 0.47 (0.44 to 0.49) in 2010.

**Fig. 4:**
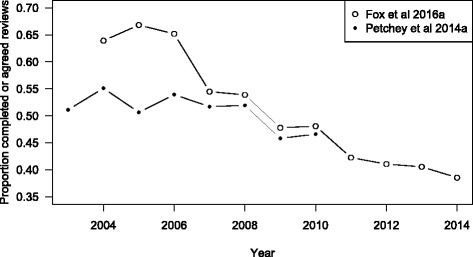
Comparison of proportion of review requests that were completed [16], *filled circles*, or agreed [5], *open circles*, for *Functional Ecology* between 2003 and 2014

A plot comparing our re-analysis of the Petchey et al. [16] data for *Functional Ecology* with that of the Fox et al. [5] data for the same journal shows consistent discrepancies (Fig. 4). Specifically, the data from Petchey et al. [16] report lower proportions across all years, with this effect most marked for the years prior to 2007 (Fig. 4).

## Discussion

Our results for the carefully collected dataset in *Molecular Ecology* found no evidence for a reduction in the proportion of completed reviews between 2009 and 2015. While the number of review invitations seemed to increase slightly over the years, this was counteracted by a larger number of completed reviews over time.

Our results on the re-analyses of data from four other ecology journals suggest that, while current reviewer completion rates are broadly similar across journals, there appears to be a declining trend in completion rates across these four journals through time. For some journals, we also noticed periods of apparent stasis in reviewer completion rate across several years followed by abrupt declines. Reviewer fatigue may have contributed to these declines; however, there are other possible explanations that are addressed below.

Differences between journals and patterns within them could be due to operational and manuscript management reasons. Decreases in the amount of time given for review turnaround may negatively affect completion rates. At *Molecular Ecology*, the turnaround time for reviews was 2 weeks for the entire period examined here. *Journal of Applied Ecology* and *Journal of Animal Ecology* had 3-week review turnaround times, which are fairly generous and these did not change over the period considered (Lindsay Haddon, pers. comm.). At *Journal of Ecology* and *Functional Ecology*, there was a switch from 3 to 2 weeks around 2009 (Lindsay Haddon, pers. comm.), so this is unlikely to explain the drop in completion rates in previous years.

Some journals may reject papers outright once a certain number of review invitations are declined, leading to lower completion rates. However, none of the journals examined here have this practice (Lindsay Haddon, pers. comm.). Finally, the number of reminders sent to reviewers may change over time impacting the completion rate. We do not have these data for the BES journals, but at *Molecular Ecology* reminders changed from two to one in early 2013 and this is not correlated with any change in completion rate (Fig. 1).

Changes in Editor identities (either the Chief Editor or Associate Editors) may affect reviewer willingness to accept or complete reviews. Researchers may be reluctant to accept review requests from particular Editors, and Editors also differ in their skill at selecting people who are likely to accept the review request. However, all journals have Editors of varying reputation and ability, and these differences should average out when data are considered among journals, particularly those with similar scope and impact factor.

Differences in how manuscripts and reviewer responses were coded across years may also contribute to differences between *Molecular Ecology* and the other journals. For example, since the review process for resubmitted manuscripts tends to be different from original submissions (original reviewers are re-invited; decisions can be made with fewer reviews), it is possible that these trends would be different, or disappear, if these datasets were restricted to original article submissions (as for the *Molecular Ecology* dataset). The timeline studied was also shorter for *Molecular Ecology*, and it is possible that examination of a longer time period would have revealed a trend, especially if the rate of decline is slow. Unfortunately, *Molecular Ecology* only adopted ScholarOne in 2008, and the data from previous years is unavailable.

**Fig. 5:**
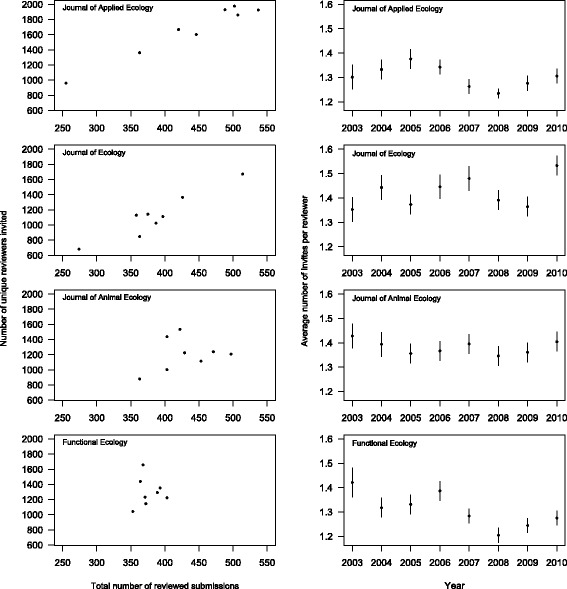
*Left column*: comparison of the number of reviewed submissions, and the number of unique reviewers invited for four ecology journals, 2003 to 2010. Data from Petchey et al. (2014b). *Right column*: comparison of the average number of review invitations per unique reviewer for four ecology journals, 2003 to 2010. *Error bars* indicate 95 % confidence intervals. Data from Petchey et al. (2014b)

Finally, perceived journal quality, and changes therein over time, could lead to changes in reviewer acceptance and completion rates. The impact factor (IF) for *Molecular Ecology* fluctuated between 5.5 and 6.5 over the study period, while the review completion rate was relatively static (Fig. 1). In contrast, the IFs of the four other journals all increased over the range of years in the datasets. Somewhat surprisingly, changes in the proportion of completed reviews seem to be negatively related to increasing impact factor (Fig. 3). A possible explanation for this relationship is that increased IF leads to increased submissions and therefore increased reviewing demands on a shrinking or static reviewer pool. This question was previously addressed for *Molecular Ecology* [19], where it was shown that as submissions increase, the pool of reviewers also increases concomitantly, such that the average number of requests per reviewer stays more or less the same. We compiled these data for the four BES journals and discovered a very similar pattern (Fig. 5). As the number of submissions increases, the number of unique reviewers invited also increases, and the average number of requests per reviewer stays fairly stable across years. This suggests that increasing requests to a shrinking pool of reviewers within journals cannot account for the drop in completion rates for *Journal of Applied Ecology*, *Journal of Ecology*, or *Journal of Animal Ecology.* However, given that the number of journals has increased through time, we cannot rule out the possibility that these reviewers are facing an increasing number of requests from other journals.

Interestingly, our results for *Functional Ecology* do not match those reported in Fox et al. [5] for this journal over the same period (2004–2010; Fig. 4). Fox et al. [5] focused on review request acceptance rate rather than review completion rate. They state that approximately 5 % of accepted reviews were not completed but make no mention of whether this rate varies between years. This difference of 5 % would account for the lower estimates of completion rate from the Petchey et al. [16] data for 2007 to 2010 but not the 10–20 % differences observed between 2004 and 2006. However, Fox et al. [5] state that non-responses to reviewer requests were not consistently recorded prior to 2007. This may account for the differences since the Petchey et al. [16] data had consistently higher estimates for the number of review invitations in the pre-2007 years than did the Fox et al. [5, 6] data.

All four other ecology journals show a trending decline in completion rate from 2007 onwards (Fig. 3), and this trend is confirmed to continue to 2014 for *Functional Ecology* [5]. The discrepancy in the pre-2007 results for *Functional Ecology* between the two datasets suggest that perhaps the data from all four journals prior to 2007 may not reliably show the proportion of review requests that led to a completed review. The comparison of the Petchey et al. [16] data with Fox et al. [5] highlights the possibility that the review request success rates have been steadily declining since 2003 at all four journals.

## Conclusions

We did not see a significant decline in review completion rate at *Molecular Ecology* from 2009 to 2015. These results differ from those at four other ecology journals, where a decline was generally observed. These differences between journals may point to subtleties in the relationship between the effort required to obtain reviews and the specifics of journal identity and management. Overall, our data suggest that reviewer agreement rates have declined at some journals but not to the dramatic extent suggested by the anecdotal and rhetorical evidence. Future research could extend this type of analysis to a broader range of journals, investigate trends over longer time periods, and include more detail about reviewer and editorial characteristics to better control for these factors when making comparisons among journals.
